# Differential Expression of Three Members of the Multidomain Adhesion CCp Family in *Babesia bigemina*, *Babesia bovis* and *Theileria equi*

**DOI:** 10.1371/journal.pone.0067765

**Published:** 2013-07-03

**Authors:** Reginaldo G. Bastos, Carlos E. Suarez, Jacob M. Laughery, Wendell C. Johnson, Massaro W. Ueti, Donald P. Knowles

**Affiliations:** 1 Department of Veterinary Microbiology and Pathology, Washington State University, Pullman, Washington, United States of America; 2 Animal Disease Research Unit, USDA Agricultural Research Service, Pullman, Washington, United States of America; Institut national de la santé et de la recherche médicale - Institut Cochin, France

## Abstract

Members of the CCp protein family have been previously described to be expressed on gametocytes of apicomplexan *Plasmodium* parasites. Knocking out *Plasmodium CCp* genes blocks the development of the parasite in the mosquito vector, making the CCp proteins potential targets for the development of a transmission-blocking vaccine. Apicomplexans *Babesia bovis* and *Babesia bigemina* are the causative agents of bovine babesiosis, and apicomplexan *Theileria equi* causes equine piroplasmosis. Bovine babesiosis and equine piroplasmosis are the most economically important parasite diseases that affect worldwide cattle and equine industries, respectively. The recent sequencing of the *B. bovis* and *T. equi* genomes has provided the opportunity to identify novel genes involved in parasite biology. Here we characterize three members of the CCp family, named CCp1, CCp2 and CCp3, in *B. bigemina*, *B. bovis* and *T. equi*. Using *B. bigemina* as an *in vitro* model, expression of all three *CCp* genes and proteins was demonstrated in temperature-induced sexual stages. Transcripts for all three *CCp* genes were found *in vivo* in blood stages of *T. equi*, and transcripts for *CCp3* were detected *in vivo* in blood stages of *B. bovis*. However, no protein expression was detected in *T. equi* blood stages or *B. bovis* blood stages or *B. bovis* tick stages. Collectively, the data demonstrated a differential pattern of expression of three orthologous genes of the multidomain adhesion CCp family by *B. bigemina*, *B. bovis* and *T. equi*. The novel CCp members represent potential targets for innovative approaches to control bovine babesiosis and equine piroplasmosis.

## Introduction

Arthropod-borne protozoan parasites of the phylum Apicomplexa cause major diseases in humans and animals, including malaria, babesiosis, and theileriosis [1], [2], [3]. Apicomplexan parasites have complex life cycle including asexual stages in vertebrate hosts and sexual stages in arthropod vectors [4]. There are currently many knowledge gaps in our understanding of the biology of these parasites, including the molecular events involved in their sexual reproduction. A better comprehension of the sexual events is needed to develop improved approaches to control the diseases caused by apicomplexan parasites. The growing number of available apicomplexan genomes has provided the opportunity to identify novel genes implicated in the parasite life cycle. Genome-based approaches have identified a highly conserved family of six multidomain adhesion proteins, termed CCp family, that are exclusively expressed on the surface of *Plasmodium* gametocytes [5], [6]. Importantly, knock out of the *Plasmodium CCp* genes blocks sporozoite formation, thus preventing the development of the parasite in the mosquito vectors, and making the CCp proteins potential targets for the development of transmission-blocking vaccines [6], [7]. The CCp proteins contain at least one signature domain *Limulus* coagulation factor C (LCCL) and additional adhesion domains, suggesting that these proteins may mediate cell contacts of gametocytes [8]. Members of the CCp family have also been identified in other apicomplexan genera, including *Toxoplasma*, *Cryptosporidium*, *Eimeria*, *Theileria* and *Ascogregarina* [9], [10]. Recently three genes of the CCp family have been identified in *Babesia divergens* [11]. Interestingly, the CCp family has not been identified in any other protozoan except apicomplexan parasites.

*Babesia bovis* and *Babesia bigemina* are tick-borne apicomplexan parasites that cause bovine babesiosis, an economically important disease of cattle that threatens the development of the bovine industry globally [12]. Similarly, the tick-borne apicomplexan parasite *Theileria equi* is the causative agent of equine piroplasmosis, the most economically important parasite disease to the horse industry worldwide [13], [14]. Extensive research efforts have concentrated on the development of approaches to control bovine babesiosis and equine piroplasmosis. However, vaccination with live attenuated parasites is still the most efficient method to prevent bovine babesiosis, and currently there is no vaccine available to control equine piroplasmosis. The recent sequencing of the *B. bovis* genome and the *T. equi* genome has provided a crucial tool to investigate the biology of these apicomplexan parasites [15], [16]. Consequently, genome-based studies have identified novel genes and gene families, resulting in a better understanding of parasite biology [17], [18]. Despite these efforts, there is a substantial lack of knowledge concerning the sexual stages of *B. bovis*, *B. bigemina* and *T. equi*. Identification of sexual stage proteins is critical to comprehend parasite biology and develop new immunological and therapeutic interventions to disrupt the parasite life cycle.

In this study we characterized three orthologous genes of the multidomain adhesion CCp family, named *CCp1*, *CCp2* and *CCp3*, in *B. bigemina*, *B. bovis* and *T. equi*. *B. bigemina* was used as an *in vitro* model for sexual stages, and expression of all three *CCp* genes and proteins was demonstrated in temperature-induced sexual stages. Additionally, the presence of *CCp* transcripts was shown *in vivo* in blood stages of *B. bovis* and *T. equi.* The newly described orthologous members of the CCp family in *B. bigemina*, *B. bovis* and *T. equi* represent rational targets for the development of novel immunological and therapeutic strategies to control bovine babesiosis and equine piroplasmosis.

## Materials and Methods

### Babesia Bigemina

*B. bigemina* isolated in Puerto Rico in 1985 was used for *in vitro* experiments and parasite culture was performed as described elsewhere [19]. The medium volume of the *B. bigemina* culture was expanded from 12 ml to 120 ml in a 5-day period, with no addition of extra red blood cells, to increase parasitemia. The expanded culture was used to induce sexual stages as previously described [20]. Briefly, to induce *B. bigemina* sexual stages, the parasite culture was incubated at 28°C in 5% CO_2_ for up to 48 h. As a control, a portion of the culture was maintained at 37°C in 5% CO_2_ for up to 48 h. A Percoll gradient was then used to separate the extracellular from intracellular parasites and the two populations were used to investigate the expression of the CCp genes and proteins. Additionally, bovine sera from *B. bigemina*-infected animals (Animal Disease Research Unit, USDA, Pullman, WA) were used as controls in immunoblot and immunofluorescence assays.

### Babesia Bovis

*B. bovis* Texas strain [21] was used in this study and parasite culture was performed as described elsewhere [22]. For the *B. bovis in vivo* studies, a splenectomized calf was intravenously inoculated with approximately 10^6^ *B. bovis*-infected erythrocytes. For tick-acquisition feeding, 1 gram of larvae (approximately 20,000 larvae) of *Rhipicephalus* (*Boophilus*) *microplus* La Minita strain [23] was placed within a cloth on a splenectomized calf. When approximately 1–2% of the ticks had molted to adult stage, the calf was inoculated with *B. bovis* as described above and the peak protozoan parasitemia occurred when the adult female ticks were reaching repletion. The calf was monitored daily for the presence of clinical signs of acute bovine babesiosis and maintained throughout the experiment in accordance to protocols approved by the University of Idaho Institutional Animal Care and Use Committee (Permit Number: 2010–54). *B. bovis* parasitemia in bovine peripheral blood and tick guts was assessed by quantitative real-time PCR (qPCR) using primers to *msa-1* gene as previously described [24].

### Theileria Equi

*T. equi* Florida isolate was used in this study and parasite culture was performed as described elsewhere [16]. For the *T. equi in vivo* studies, a spleen-intact horse was inoculated intravenously with approximately 8×10^7^ infected erythrocytes. The horse was monitored daily for the presence of clinical signs of acute equine piroplasmosis and maintained throughout the experiment in accordance to protocols approved by the University of Idaho Institutional Animal Care and Use Committee (Permit Number: 2010–54). *T. equi* parasitemia in horse peripheral blood was assessed by qPCR using primers to *ema-1* gene as previously described [25].

### *In silico* Gene Identification

*In silico* gene identification was performed by comparing the amino acid sequences of CCp1 (GenBank ID XP_001348897.1) and CCp3 (GenBank ID XP_001348240) of *P. falciparum* 3D7 strain, and CCp2 (GenBank ID XP_001615829) of *P. vivax* Sal-1 strain to the *B. bovis* and *T. equi* genomes using BLAST software (http://blast.ncbi.nlm.nih.gov/Blast.cgi). *In silico* gene identification was also accomplished by comparing the amino acid sequences of *B. divergens CCp* genes [11] to the *B. bovis* and *T. equi* genomes. Amino acid identity ≥ than 30% was used as a criterion for identification of the CCp orthologous genes in *B. bovis* and *T. equi*. Multiple alignments of amino acid sequences were generated using the Multiple Alignment Module of LaserGene (http://www.dnastar.com). *In silico* analysis of the available *B. bigemina* sequences was performed using BLASTN and TBLAST at the Sanger Institute website (http://www.sanger.ac.uk/cgi-bin/blast/submitblast/b_bigemina). The Simple Modular Architecture Research Tool (SMART) (http://smart.embl-heidelberg.de) and the Transmembrane Hidden Markov Model package 2 (TMHMM2) (http://www.cbs.dtu.dk/services/TMHMM-2.0) were used to predict domains and signal peptides in the CCp protein sequences.

### RNA Isolation and Transcript Level Analysis

Reverse transcriptase quantitative real-time PCR (RT-qPCR) was standardized to assess the level of expression of the *CCp1*-*3* genes in *B. bovis*, *B. bigemina* and *T. equi*. Parasite cultures, infected blood and tick guts were collected in RNAlater® (Ambion) and stored at −20°C following the manufacture’s protocol. Total RNA was extracted using the RNAqueous® Kit (Ambion) according to the manufacturer’s protocol. The total RNA samples were analyzed by the Experion™ Automated Electrophoresis System (Bio-Rad) and only samples with an RNA Quality Indicator (RQI) ≥ than 7 were used for cDNA synthesis (Figure S1). Two hundred nanograms of total RNA were utilized for cDNA synthesis using the Superscript® Vilo™ cDNA Synthesis Kit (Invitrogen) following the manufacturer’s protocol. The RT-qPCR were performed in a CFX96™ Real-Time PCR Detection System using the SsoFast™ EvaGreen® Supermix (Bio-Rad). The cycling conditions consisted of an enzyme activation step of 95°C for 30 seconds followed by 40 cycles of 95°C denaturation for 5 seconds and annealing/extension of 60°C for 5 seconds. Reactions were performed in duplicate in 20 µl using 200 nM of each primer and 2 µl of a 1/20 dilution of cDNA as template. The CFX Manager™ Software (Bio-Rad) was used to analyze the RT-qPCR data. Gene expression was normalized to the total amount of RNA used to generate the cDNA, as previously described [24]. The transcription level was then calculated as a relative expression using the formula: Relative expression _(sample)_ = 2 ^[C*q* (control) – C*q* (sample)]^, where the control is the highest C*q* value for a given gene of interest, as previously described [24]. Efficiency of amplification and melt curve analyses was performed to evaluate analytical sensitivity and specificity of the RT-qPCR for each gene of interest (Table S1). Standard PCR for bovine actin (primers: gtgtggattggcggct and tactcctgcttgctgat) and horse actin [26] were performed to demonstrate the presence of amplifiable cDNA in all tested samples.

### Synthetic Peptides and Polyclonal Antibodies

**Figure 1:**
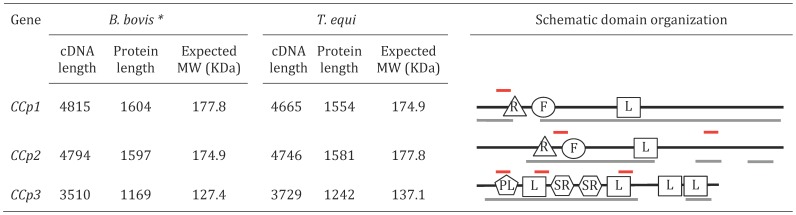
Gene identification, cDNA length, protein length, expected protein size and schematic domain representation of CCp1-3 in *Babesia bovis* and *Theileria equi*. Black lines represent the full-length of the CCp proteins in *B. bovis* and *T. equi*. Grey lines represent the regions of the CCp proteins available in *B. bigemina*. Red lines above CCp1-3 indicate the regions used to design synthetic peptides. R: Ricin B lectin domain. F: F5/F8 type C domain. L: LCCL domain. PL: PLAT domain. SR: Scavenger receptor domain. * *In silico* data of *B. bovis* CCp1-3 has been previously shown by Becker *et a*l [11].

Synthetic peptides ranging from 15 to 19 amino acids were produced based on the sequence of the *B. bovis* CCp proteins, as follows: CCp1– KTFESKPSYKEVFK (aa 122–135); CCp2.1 - ESAKKTKDARDKYFLQSV (aa 530–547) and CCp2.2 KRLIRVVNGDPYEIAKIED (aa 1165–1183); and CCp3.1 - PSSLKGTYIYTEDSSI (aa 622–637), CCp3.2 GSYLTFVVEAADVGDVNGI (aa 123–141) and CCp3.3 ASAMFDGVLTPSGGE (aa 254–268) (Figure 1, Table S2, Figure S2, Figure S3 and Figure S4). The percentage of amino acid identity of the peptides and the *B. bigemina* and *T. equi* CCp sequences is shown in Table S2. Three 6-week old BALB/c female mice were inoculated with 100 µg of KLH-conjugated CCp1 peptide emulsified in TiterMax® Gold Adjuvant (Sigma) followed by six booster inoculations at 2 weeks interval. Serum titers were assessed by ELISA and the mice exsanguinated 7 days after the last booster inoculation. Two groups of two rabbits were inoculated with either KLH-conjugated CCp2 peptides or KLH-conjugated CCp3 peptides followed by four booster inoculations at 2 weeks interval following the protocol described by Bio-Synthesis, Inc (http://www.biosyn.com). Serum titers were assessed by ELISA and the rabbits exsanguinated 7 days after the last booster inoculation. The rabbit and mouse polyclonal antibodies were used in immunoblot and immunofluorescence assays to investigate the expression of the CCp proteins.

### Immunoblot Assays

Antigens for immunoblot were prepared from *B. bigemina* culture, *B. bovis*-infected bovine blood, *B. bovis*-infected tick guts, or *T. equi*-infected horse blood. After adding Laemmli buffer (Bio-Rad), the samples were boiled for 10 min and approximately 10 µg of total protein were separated in 4–20% Mini-PROTEAN® TGX™ Precast Gels (Bio-Rad). The proteins were transferred overnight at 4°C to nitrocellulose membranes. The membranes were blocked for non-specific binding in TNT (10 mM Tris, 150 mM NaCl, and 0.05% Tween 20) containing 5% non-fat milk for 1 hour at room temperature (RT). The membranes were then incubated for 1 hour at RT with an appropriate primary antibody. After washing in TNT, the membranes were incubated with an appropriate HRP conjugated secondary antibody for 30 minutes at RT. After washing in TNT, the immunoblot assays were developed using chemiluminescent HRP antibody detection reagents.

### Immunofluorescence Assays

Immunofluorescence assays were performed using antigens from *B. bigemina* culture, *B. bovis*-infected bovine blood, *B. bovis*-infected tick guts, or *T. equi*-infected blood. *B. bigemina* culture and parasite-infected blood were adjusted to 20% pack cell volume (PCV) in PBS containing 1% BSA. Ticks fed on a *B. bovis*-infected calf were dissected and individual tick guts were suspended in PBS containing 1% BSA. Approximately 5 µl of either the 20% PCV solution or the tick gut suspension was used to make a uniform thin film on glass microscope slides. The slides were air dried and fixed for 1 minute in cold acetone. The slides were incubated at 37°C in a humidity chamber for 30 minutes with different concentrations of an appropriate primary antibody. After washing in PBS, the slides were incubated at 37°C in a humidity chamber for 30 minutes with an appropriate FITC conjugated secondary antibody. After washing in PBS, the slides were examined using an epifluorescence microscope equipped with FITC UV excitation and emission filters.

## Results

### *In silico* Identification of the *CCp* Genes

Three orthologous members of the *CCp* gene family, termed *CCp1-3*, were identified by comparing the *CCp* sequences of *Plasmodium spp.* to the *B. bovis* and *T. equi* genomes (Figure 1 and Table S3). The genome screening analysis indicated that orthologous *CCp1*-*3* are single copy genes in the *T. equi* genome and, as previously described in the *B. bovis* genome [11]. In the *B. bovis* genome, CCp1-3 proteins are identified as BBOV_III006360, BBOV_II003700 and BBOV_III008930, respectively, and are annotated as LCCL domain containing proteins. In the *T. equi* genome, CCp1-3 are identified as BEWA_020400, BEWA_004750 and BEWA_010590, respectively, and annotated as bromodomain containing protein, hypothetical protein and LCCL domain-containing protein, respectively. It was predicted that *B. bovis CCp1*-*3* genes encode for proteins with molecular weight of 177.8, 174.9 and 127.4 KDa, respectively, as previously described [11]. Similarly, it was predicted that *T. equi CCp1*-3 genes encode for proteins with molecular weight of 174.9, 177.8 and 137.1 KDa, respectively. No orthologous genes for *CCp4* and *CCp5* were found in the *B. bovis* and *T. equi* genomes. Analysis of the available *B. bigemina* genome sequences also revealed the presence of *CCp1*-*3* in this apicomplexan parasite. However, no full-length sequence of the *CCp* genes was obtained from *B. bigemina*, as the complete genome sequence for this parasite is not yet available. Pairwise analysis of the amino acid sequence of CCp1-3 of *B. bovis*, *T. equi*, *B. divergens* and *P. falciparum* revealed the level of interspecies identity and similarity of these proteins (Table S4). Taken together, the *B. bovis* CCp1-3 proteins showed greater identity and similarity to *B. divergens* than to the *T. equi* and *P. falciparum* sequences. Likewise, the *T. equi* CCp1-3 sequences showed a higher level of identity and similarity to *B. bovis* than to *P. falciparum*. It is noteworthy that the amino acid identity and similarity was distributed throughout the length of the CCp proteins of *B. bovis*, *T. equi* and *Plasmodium spp.* (Figure S2, Figure S3 and Figure S4). Regarding domain organization, the *B. bovis* and *T. equi* CCp proteins presented similar domain distribution when compared to orthologous members in other apicomplexan parasites. CCp1 and CCp2, both in *B. bovis* and *T. equi*, contain a similar domain pattern with one LCCL domain, one ricin B lectin domain and one F5/8 type C domain. CCp3 protein, both in *B. bovis* and *T. equi*, presents three LCCL domains, two scavenger domains and one PLAT (polycystin-1, lipoxygenase, alpha-toxin) domain (Figure 1).

### Expression of *CCp1-3* Genes in *Babesia bigemina*

**Figure 2:**
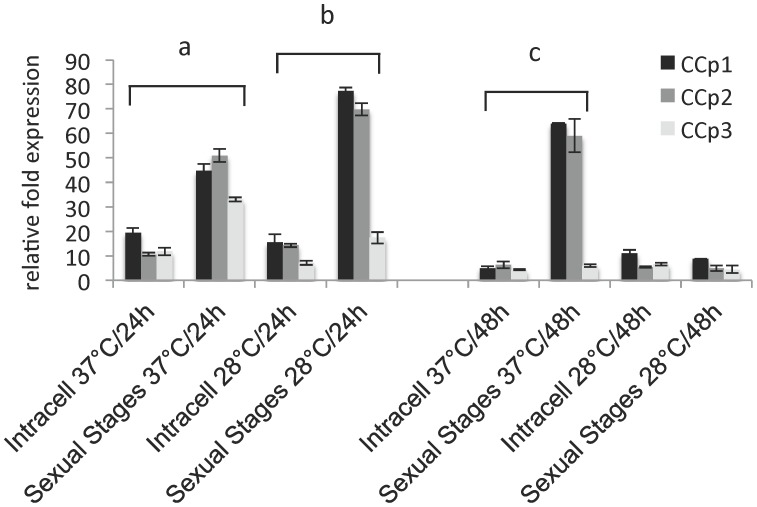
Relative expression of *B. bigemina CCp1-3* by extracellular sexual stages incubated either at 37°C or 28°C for 24 h and 48 h is compared to gene expression by intracellular parasites (Intracell) incubated under identical conditions for each temperature and time point. Gene expression was normalized to the total amount of RNA used to generate the cDNA. The transcription level was calculated as a relative expression using the formula: Relative expression _(sample)_ = 2 ^[C*q* (control) – C*q* (sample)]^, where the control is the highest C*q* value for a given gene of interest. Results represent the means of three experiments, each containing three technical replicates. Differences in gene expression were determined by the Student’s *t*-test. “a” indicates the expression of *CCp1*-*3* was significantly higher (P<0.001) by sexual stages incubated for 24 h at 37°C than by Intracell. “b” indicates the expression of *CCp1*and *CCp2* was significantly higher (P<0.001) by sexual stages incubated for 24 h at 28°C than by Intracell. “c” indicates the expression of *CCp1*and *CCp2* was significantly higher (P<0.001) by sexual stages incubated for 37 h at 48°C than by Intracell.

We initially examined the expression of the *CCp* genes in *B. bigemina*, since this is the only experimental *in vitro* system currently available to investigate sexual stages in *Babesia spp.* and *Theileria spp.* [4], [20], [27]. *B. bigemina* sexual stages were induced by incubating the parasite culture for up to 48 h at 28°C in 5% CO_2_. The extracellular temperature-induced sexual stage parasites were used to investigate the level of expression of the *CCp* genes and proteins. Extracellular *B. bigemina* sexual stage parasites were separated from intraerythrocytic parasites by Percoll gradient. Total RNA was extracted from the two parasite populations and relative expression of *CCp1*-*3* was accessed by RT-qPCR (Figure 2). The extracellular population of *B. bigemina* sexual stages showed a significant up regulation of all three *CCp* genes when compared to intracellular parasites. Expression of *CCp1* and *CCp2* peaked at 24 h in sexual stages incubated at 28**°**C and at 48 h in sexual stages incubated at 37**°**C. In contrast, *CCp3* peaked at 24 h in sexual stages incubated at 37**°**C. After 48 h of incubation, there was no difference in the expression of *CCp3* in sexual stages incubated at either 37**°**C or 28**°**C. It is noteworthy that the normalized expression of *CCp1* and *CCp2* increased (P<0.001) more than 40-fold in sexual stages incubated at 28**°**C for 24 h when compared to intracellular parasites incubated at the same condition (Figure 2). The results presented in Figure 2 represent the means of three experiments, each containing three technical replicates. It is important to mention that despite the up regulation of *CCp-1-3* genes in *B. bigemina* sexual stages, transcripts for all the three genes were also detected in non-induced parasite cultures kept at 37°C (Figure S5).

### Expression of CCp1-3 Proteins in *Babesia bigemina*

**Figure 3:**
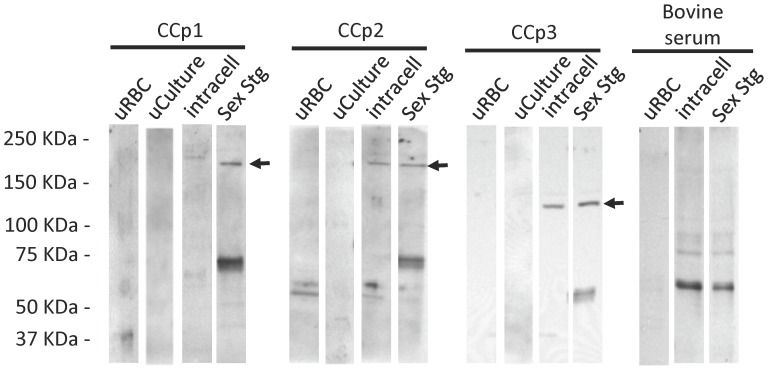
Immunoblot analysis demonstrating the expression of CCp1-3 by temperature-induced *B. bigemina* parasites. Expression of CCp1-3 proteins was investigated in *B. bigemina* cultures incubated for 24 h at 28°C. Expression of CCp1 was detected only by extracellular sexual stages (Sex Stg) whereas CCp2 and CCp3 were detected in both Sex Stg and intracellular parasites (Intracell) populations. No bands with the expected molecular mass of the CCp proteins were detected in non-induced *B. bigemina* cultures (uCulture) or in uninfected red blood cells (uRBC). Polyclonal mouse anti-CCp1 peptide, rabbit anti-CCp2-peptides or CCp3-peptides, and bovine serum from an animal chronically infected with *B. bigemina* were used at a 1∶50 dilution. Anti-mouse, anti-rabbit, or anti-bovine HRP conjugates were used at a 1∶4,000 dilution. Black arrows indicate the expected size of CCp1-3.

**Figure 4:**
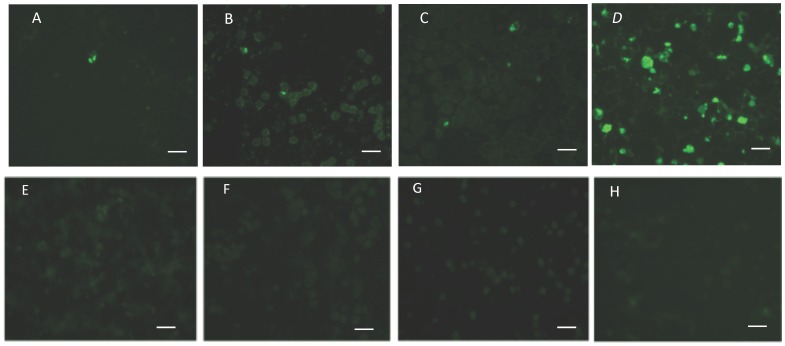
Immunofluorescence assays demonstrating the expression of CCp1-3 in temperature-induced *B. bigemina* cultures. Antigen for immunofluorescence was prepared from parasite culture adjusted to 20% pack cell volume (PCV). Five µl of the 20% PCV solution was used to make a uniform thin film on glass microscope slides. Expression of CCp1-3 proteins in temperature-induced *B. bigemina* gametocyte cultures incubated for 24 h at 28°C is shown in panels A, B and C, respectively. CCp expression was not detected in non-induced *B. bigemina* cultures kept at 37°C as shown in panels E, F, and G. Panels D and H are representative results of immunofluorescence using sera from either *B. bigemina*-infected or uninfected bovine, respectively. Polyclonal mouse anti-CCp1 peptide, rabbit anti-CCp2 or CCp3 peptides, and bovine sera were used at a 1∶20 dilution. Anti-mouse, anti-rabbit, or anti-bovine FITC conjugates were used at a 1∶80 dilution. The fields shown are representative of the majority of the samples. The panels present a magnification of 100x and white bars indicate 10 µm.

Expression of CCp1-3 proteins was demonstrated *in vitro* in temperature-induced *B. bigemina* sexual stages (Figure 3 and 4). Immunoblot analysis using mouse polyclonal anti-CCp1 peptide detected the expression of the CCp1 protein by *B. bigemina* extracellular sexual stages; however no band with the putative expected size of CCp1 was visualized in intracellular parasites. Interestingly, rabbit polyclonal antibodies against either CCp2 peptides or CCp3 peptides detected the expression of CCp2 and CCp3, respectively, from both extracellular sexual stages and intracellular *B. bigemina*. No expression of CCp proteins was detected by non-induced *B. bigemina* cultures using immunoblot (Figure 3). Bovine sera from *B. bigemina* chronically infected animals reacted primarily to the 58 KDa Rap-1 protein using both intracellular parasites and extracellular sexual stages. The *B. bigemina*-infected bovine sera also detected other antigens with molecular mass ranging from 75 to 90 KDa. However, the tested *B. bigemina*-infected bovine sera did not detect antigens with the expected size of the CCp proteins. Figure 3 shows an immunoblot using a representative *B. bigemina*-infected bovine serum. Immunofluorescence assays using mouse and rabbit polyclonal antibodies generated against CCp synthetic peptides revealed the expression of CCp1-3 in temperature-induced *B. bigemina* cultures (Figure 4). No expression of CCp proteins was detected by non-induced *B. bigemina* cultures using immunofluorescence (Figure 4). Immunoblot results showed CCp2 and CCp3 expression by both extracellular and intracellular *B. bigemina* (Figure 3), and consequently, the immunofluorescence assays were performed using whole culture without Percoll gradient separation. The parasitemia of the *B. bigemina* culture used for immunofluorescence antigen was approximately 8–10%. With respect to the CCp proteins, only one or two fluorescent parasites were identified on average per microscopic field (100x magnification) indicating that a very low percentage of the temperature-induced *B. bigemina* parasites were expressing the proteins (Figure 4). Negative controls for the immunofluorescence assay, including pre-immune sera, FITC conjugate, and normal bovine serum are shown in Figure S6.

### Expression of CCp1-3 in Babesia Bovis

**Figure 5:**
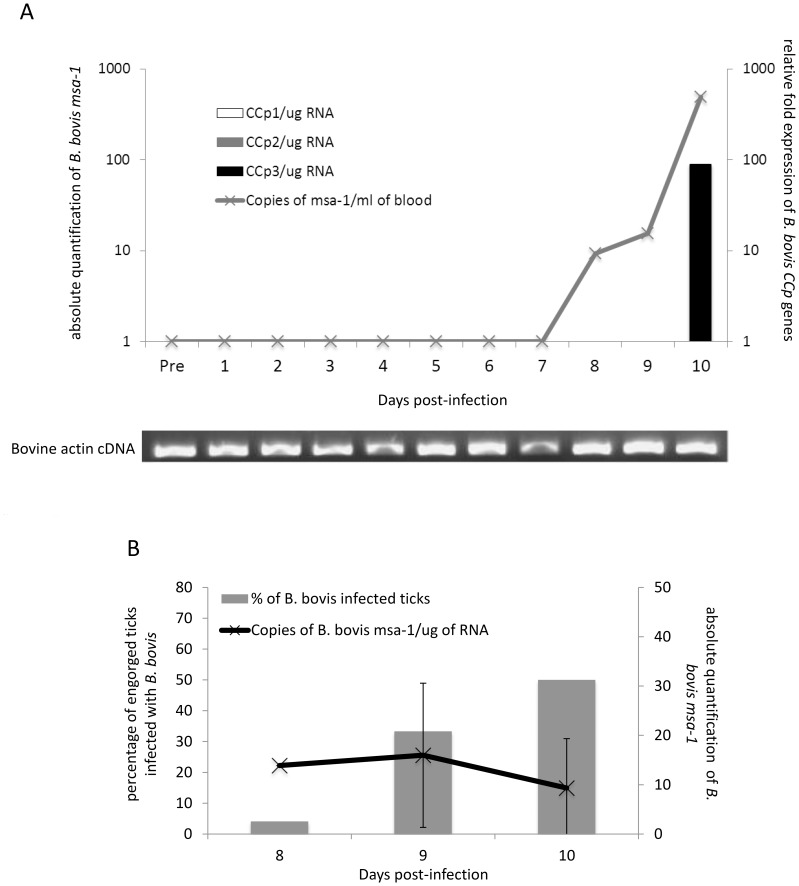
Expression of *CCp1*-*3* during acute *B. bovis* infection. In panel A, relative fold expression of *CCp1-3* transcripts in bovine blood during acute *B. bovis* infection is shown in the right y-axis. Absolute quantification of *B. bovis msa-1* DNA in bovine blood is shown in the left y-axis. *CCp* gene expression was normalized to the total amount of RNA used to generate the cDNA and the transcription level was calculated as a relative expression using the formula: Relative expression _(sample)_ = 2 ^[C*q* (control) – C*q* (sample)]^, where the control is the highest C*q* value for a given gene of interest. To confirm the presence of amplifiable cDNA in all tested samples a standard PCR for bovine actin was performed. In panel B, absolute expression of *B. bovis msa-1* RNA in bovine blood is shown in the right y-axis and the percentage of engorged ticks infected with *B. bovis* is shown in the left y-axis.

Expression of *CCp1*-*3* genes was evaluated *in vitro* in *B. bovis* culture, and *in vivo* in cattle during acute infection and ticks fed on a calf during acute infection. Transcripts for all three *CCp* genes were detected in *B. bovis* culture (Figure S5). During the acute phase of *B. bovis* infection, the calf temperature ranged from 38.1 to 41.3°C and PVC decreased approximately 40%. *B. bovis* parasites were first detected at day 8 post-infection and the peak parasitemia (approximately 500 parasites/ml of blood) occurred at day 10 post-infection (Figure 5A). At that time, the *B. bovis*-infected calf had to be euthanized due to the severity of the disease. Relative expression of the *B. bovis CCp* genes was evaluated in the calf blood during acute infection. Expression of the *B. bovis CCp3* gene was detected at day 10 post-infection. Interestingly, *B. bovis CCp1* and *CCp2* were not detected during the evaluation period. Standard PCR for bovine actin was performed to demonstrate the presence of amplifiable cDNA in all tested samples (Figure 5A). It is noteworthy that no expression of *B. bovis* CCp proteins was detected by immunoblot or immunofluorescence either in parasite culture or bovine blood during acute infection. The presence of *B. bovis* DNA was also examined in engorged *R. (B.) microplus* female ticks fed on a calf during acute infection. At days 8, 9 and 10 post-*B. bovis* infection, 24 engorged *R. (B.) microplus* females were collected per each time point. The ticks were dissected and guts collected in PBS for genomic DNA extraction and in RNAlater® for RNA extraction. A timeframe of less than 2 h passed between tick collection/dissection and tick guts storage in RNAlater®. Genomic DNA and total RNA were extracted from individual guts of ticks fed on the *B. bovis*-infected calf, and qPCR and RT-qPCR for *msa-1* gene were performed. At day 8 of feeding, only one tick gut was positive for *B. bovis* and the positive sample had 200 parasites per µg of gut DNA. At day 9 of feeding, 33.3% of the tick guts (9 out of 24) were positive for *B. bovis* and the average number of parasites was 196 (±21) per µg of tick gut DNA. At day 10 of feeding, 50% of the tick guts (12 out of 24) were positive for *B. bovis* and the average number of parasites was 176 (±26) per µg of gut DNA (Figure 5B). Gene expression was examined in cDNA samples from individual guts of engorged *R. (B.) microplus* female ticks fed on a calf during *B. bovis* acute infection. As described for the genomic DNA analysis, at days 8, 9 and 10 post-*B. bovis* infection, 24 engorged *R. (B.) microplus* females were collected per each time point and cDNA synthesized from individual ticks guts. Absolute quantification of *B. bovis msa-1* transcripts was performed and ranged from 9 to 15 copies in gut samples of engorged female ticks fed on the infected calf (Figure 5B). However, no expression of *CCp1*-*3* genes was detected in the cDNA samples of tick guts. Additionally, it is noteworthy that no expression of the *B. bovis* CCp proteins was detected by either immunoblot or immunofluorescence in guts of ticks fed on a calf during acute infection.

### Expression of CCp1-3 in Theileria Equi

**Figure 6:**
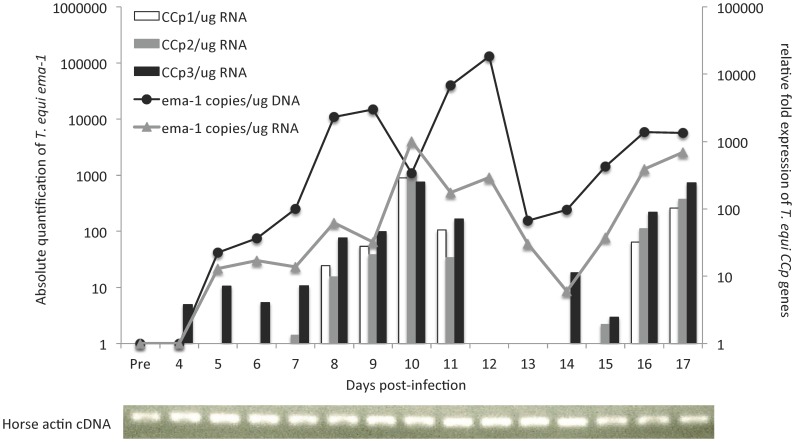
Expression of *CCp1*-*3* during acute *T. equi* infection. Relative fold expression of *CCp1-3* transcripts in horse peripheral blood during acute *T. equi* infection is shown in the right y-axis. Absolute quantification of *T. equi emai-1* DNA (black line) and cDNA (grey line) is shown in the left y-axis. *CCp* gene expression was normalized to the total amount of RNA used to generate the cDNA and the transcription level was calculated as a relative expression using the formula: Relative expression _(sample)_ = 2 ^[C*q* (control) – C*q* (sample)]^, where the control is the highest C*q* value for a given gene of interest. To confirm the presence of amplifiable cDNA in all tested samples a standard PCR for horse actin was performed.

Expression of *CCp1*-*3* genes was evaluated *in vitro* in *T. equi* culture, and *in vivo* in horse blood during acute infection. Transcripts for all three *CCp* genes were detected in *T. equi* cultures (Figure S5). During *T. equi* acute infection, the horse temperature ranged from 37.1 to 40.8°C and PCV decreased 40%. *T. equi* parasites were first detected at day 5 post-infection and the peak parasitemia (approximately 500 parasites/µg DNA) occurred at day 12 post-infection (Figure 6). Total RNA was extracted from peripheral blood of the infected horse and used to synthesize cDNA. Relative expression of the *CCp* genes was evaluated in *T. equi* blood stages during acute infection. Expression of *T. equi CCp3* was first detected at day 4 post-infection and was followed by the expression of *CCp2* and *CCp1* at days 7 and 8 post-infection, respectively. The expression of all *T. equi CCp* genes peaked at day 10 post-infection and showed an increase of more than 250-fold compared to previous days of infection. Interestingly, the *CCp* genes were not constitutively expressed during *T. equi* acute infection. In fact, during the evaluation period, the genes presented two waves of expression that began with the expression of the *CCp3* gene followed sequentially by *CCp2* and then *CCp1* genes. Expression of the *T. equi ema-1* gene was also evaluated and shown to be constitutively expressed during the acute phase of the infection. Standard PCR for horse actin was performed to demonstrate the presence of amplifiable cDNA in all tested samples (Figure 6). It is noteworthy that no expression of the *T. equi* CCp proteins was detected by immunoblot or immunofluorescence either in parasite culture or in horse blood during acute infection.

## Discussion

In this study we investigated the pattern of expression of three newly identified orthologous genes of the multidomain adhesion protein family CCp, named *CCp1*, *CCp2* and *CCp3*, by the apicomplexan parasites *Babesia bovis*, *Babesia bigemina* and *Theileria equi*. Members of the CCp family have been previously identified in *Plasmodium, Toxoplasma*, *Cryptosporidium*, *Eimeria*, *Theileria* and *Ascogregarina* [6], [8], [9]. Recently, three members of the CCp family have been identified in *B. divergens* [11], [28]; however, no biological data were previously available for *CCp* genes in *B. bovis*, *B. bigemina* or *T. equi*. Here we have demonstrated that expression of the *CCp1-3* genes was significantly up regulated *in vitro* in *B. bigemina* sexual stages. We also demonstrated a differential expression of the CCp1-3 proteins *in vitro* in *B. bigemina* sexual stages. Additionally, *CCp* transcripts were detected *in vivo* in blood stages of *B. bovis* and *T. equi* during acute infection.

*CCp1*-*3* were identified as single copy genes in the genomes of *B. bovis* and *T. equi*, as previously described for the *CCp* ortologous genes in *Plasmodium spp.* and *B. divergens* [5], [6], [11]. The presence of *CCp* orthologous genes in several apicomplexan parasites, including *Plasmodium, Toxoplasma*, *Cryptosporidium*, *Eimeria*, *Theileria*, *Ascogregarina* and *Babesia*, suggests a potentially conserved function for this family. It has been described that CCp proteins are expressed on the surface of *Plasmodium* gametocytes where they play a role in parasite interaction during zygote formation [6], [7], [8]. Therefore, it is tempting to consider that CCp proteins might play a similar function in *Babesia* and *Theileria* transmission. Development of mutant *Babesia* and *Theileria* lacking the expression of the CCp genes is essential to address this hypothesis. The presence of multi-adhesion domains and the remarkable similarity of domain organization of CCp1-3 among different apicomplexan parasites, including *B. bovis*, *B. bigemina* and *T. equi* described in this manuscript, also prompts us to argue about a potential evolutionary conserved function for these proteins. The similar size and domain organization of CCp1 and CCp2 in numerous apicomplexan parasites, including *B. bovis*, *B. bigemina* and *T. equi* presented in this study, suggests that a gene duplication event may have occurred in the common ancestor of the Apicomplexa phylum, as mentioned elsewhere [6]. Interestingly, neither *CCp4* nor *CCp5* orthologous genes were found in *B. bovis* or *T. equi*. Similar absence of both CCp members was also previously reported in *T. parva, T. annulata*, and *B. divergens* [11], [29], [30]. It has been shown in *Plasmodium* that the other member of the CCp family, termed *FNPA* gene, does not contain the LCCL signature domain [6], [10], and was not a subject of investigation in this study.

*B. bigemina* is the only experimental *in vitro* system currently available to investigate sexual stages in *Babesia* species and several studies have used this model to study sexual stages [4], [20], [27]. *B. bigemina* is a particularly important model to study sexual stages *in vitro* considering the technical limitations to investigate these stages in *Babesia* and *Theileria* species. Thus, using *B. bigemina*, we demonstrated significant up regulation of *CCp1-3* genes in temperature-induced sexual stages. We also demonstrated expression of CCp2 and CCp3 proteins both in intracellular and extracellular temperature-induced *B. bigemina*, and CCp1 expression only in extracellular temperature-induced parasites. Expression of CCp proteins in intracellular and extracellular gametocyte-committed *Plasmodium* has also been previously described [31]. It remains to be determined if the intracellular *B. bigemina* parasites expressing CCp proteins were committed to sexual stage.

It has been shown that individuals naturally infected with *Plasmodium* develop low and inconsistent immune response against the sexual stage antigens Pfs230 and Pfs48/45 [32]. In addition, no immune response against *Plasmodium* CCp proteins has been reported to date. Here we show that sera from *B. bigemina*-infected cattle do not recognize antigens in the range of the expected molecular mass of CCp proteins, suggesting low or absence of exposure of these antigens during bovine infection. This result makes the CCp proteins rational targets for the development of immunological approaches to prevent parasite transmission.

Regulation of gene expression and translational repression mechanisms play important roles in sexual differentiation and gametogenesis in eukaryotes. We showed that the *CCp1-3* genes were not constitutively expressed in *B. bovis* and *T. equi* during acute infection, suggesting the presence of a mechanism to regulate the *in vivo* expression of these genes. It has been demonstrated that post-transcriptional gene regulation is critical for *Plasmodium* zygote development [33], [34]. Additionally, a mechanism based on the RNA helicase DOZI (development of zygote inhibited) has been recently shown to repress translation in *Plasmodium* [35]. Here we document the presence of *CCp3* transcripts in *B. bovis* blood stages and *CCp1-3* transcripts in *T. equi* blood stages; however, no protein products were detected. Considering the molecular similarities among apicomplexan parasites, it is appealing to argue that a DOZI-like mechanism may be involved in translational repression in *B. bovis* and *T. equi*. Pradel et al. [31] showed that disruption of the *P. falciparum CCp3* gene leads to the loss of the expression of CCp1 and CCp2 proteins, demonstrating co-dependent expression of these genes. Consistent with this observation, we demonstrated that *in vivo* the expression of the *CCp3* gene was followed sequentially by the expression of *CCp2* and *CCp1*, respectively. This was particularly evident in the two waves of expression of the *CCp* genes during the *T. equi* acute infection, suggesting some level of co-dependent gene expression.

Control of gene expression and synthesis of stage-specific proteins have been previously demonstrated in *Plasmodium* [34], [36]. Regulation of the *B. bigemina Rap-1b* and *Rap-1c* genes has also been previously demonstrated both at the transcriptional and translational levels [37]. As mentioned above, despite the presence of transcripts, we were not able to demonstrate the expression of the CCp proteins by *B. bovis* or *T. equi* during acute infection. Additionally, *B. bovis* CCp transcripts and protein expression were not detected in guts of ticks fed on a calf during acute infection. The low number of parasites detected in tick guts and the absence of detectable CCp transcripts and proteins may reinforce the hypothesis that a low number of *B. bovis* parasites survive inside the tick vector and converge to sexual stages, as previously described in different species of *Plasmodium* [38].

In summary, in this study we investigated the pattern of expression of three orthologous genes of the highly conserved family of multidomain adhesion CCp proteins, named *CCp1-3,* by *B. bovis*, *B. bigemina* and *T. equi*. Transcripts for *CCp1*-*3* were significantly up regulated *in vitro* in temperature-induced *B. bigemina* sexual stages. Expression of all three CCp proteins was demonstrated *in vitro* in temperature-induced *B. bigemina* cultures. Gene transcripts were detected in blood stages of *B. bovis* and *T. equi*. However, no protein expression was detected in *T. equi* blood stages or *B. bovis* blood stages or *B. bovis* tick stages. It has been demonstrated in *Plasmodium* that CCp proteins are expressed on the surface of gametocytes and that the knock out of the *CCp* genes blocks parasite transmission [6], [7], [39]. It was beyond the scope of this study to investigate the function of *CCp* genes, but considering the *in silico*, *in vivo* and *in vitro* data presented in this paper, it is rational to cogitate that the orthologous members of the CCp family may play a role in sexual stages of *B. bovis*, *B. bigemina* and *T. equi*. Consequently, it is tempting to consider CCp proteins as potential targets to interrupt parasite transmission. Future studies are needed to design novel immunological and therapeutic strategies to address this hypothesis.

## Supporting Information

Figure S1 **Total RNA samples were analyzed by the Experion Automated Electrophoresis System (Bio-Rad).** Panel A shows a typical electropherogram of total RNA samples from *Babesia bovis*-infected bovine blood (The relative positions of 18S rRNA and 28S rRNA are indicated). Panel B shows a typical microfluidic electrophoresis of four representative RNA samples (1 to 4) and a molecular marker (M). The four representative samples presented RNA Quality Indicator (RQI) ≥ than 7 when analyzed by the Experion software, and in this study, only samples with RQI ≥ than 7 were used for cDNA synthesis.
(TIFF)

Figure S2 **Multiple sequence alignments of CCp1 by CLUSTALW2.** CCp2 amino acid sequences from *Babesia bovis* (Texas strain), *Theileira equi* (Florida isolate), *Plasmodium falciparum* (3D7 strain) and *Plasmodium vivax* (Sal-1 strain) were analyzed. Red line indicates the predicted location of the LCCL signature domains of the CCp protein family. Green line indicates the regions used to design synthetic peptides.
(TIF)

Figure S3 **Multiple sequence alignments of CCp2 by CLUSTALW2.** CCp3 amino acid sequences from *Babesia bovis* (Texas strain), *Theileira equi* (Florida isolate), *Plasmodium falciparum* (3D7 strain) and *Plasmodium vivax* (Sal-1 strain) were analyzed. Red line indicates the predicted location of the LCCL signature domains of the CCp protein family. Green lines indicate the regions used to design synthetic peptides.
(TIF)

Figure S4 **Multiple sequence alignments of CCp2 by CLUSTALW2.** CCp3 amino acid sequences from *Babesia bovis* (Texas strain), *Theileira equi* (Florida isolate), *Plasmodium falciparum* (3D7 strain) and *Plasmodium vivax* (Sal-1 strain) were analyzed. Red lines indicate the predicted location of the LCCL signature domains of the CCp protein family. Green lines indicate the regions used to design synthetic peptides.
(TIF)

Figure S5 **Presence of** ***CCp1*** **-** ***3*** **transcripts in cultures of** ***Babesia bovis*** **,** ***Babesia bigemina*** **and** ***Theileria equi*** **kept at 37**°**C.** Results without reverse transcriptase (RT-) are shown for each gene and parasite.
(TIFF)

Figure S6 **Immunofluorescence assays of pre-immune sera, normal bovine serum, anti-mouse FITC conjugate, anti-rabbit FITC conjugate, and anti-bovine FITC conjugate.** Mouse and rabbit pre-immune sera were used at a 1∶20 dilution. Normal bovine sera were used at a 1∶20 dilution. Anti-mouse, anti-rabbit, or anti-bovine FITC conjugates were used at a 1∶80 dilution. The fields shown here are representative of the majority of the samples. The panels present a magnification of 100x and white bars indicate 10 µm.
(TIFF)

Table S1 **Gene name, primer sequence, amplicon size and parameters of PCR efficiency of the** ***CCp*** **genes in** ***Babesia bovis*** **(** ***Bb*** **),** ***Babesia bigemina*** **(** ***Bbg*** **) and** ***Theileria equi*** **(** ***Te*** **).**
(DOC)
