# Supplementary figures and images for: Differential Expression of Three Members of the Multidomain Adhesion CCp Family in Babesia bigemina, Babesia bovis and Theileria equi
Source: PLoS One. 2013 Jul 3;8(7):e67765. doi: 10.1371/journal.pone.0067765 (PMC3701008; doi:10.1371/journal.pone.0067765)

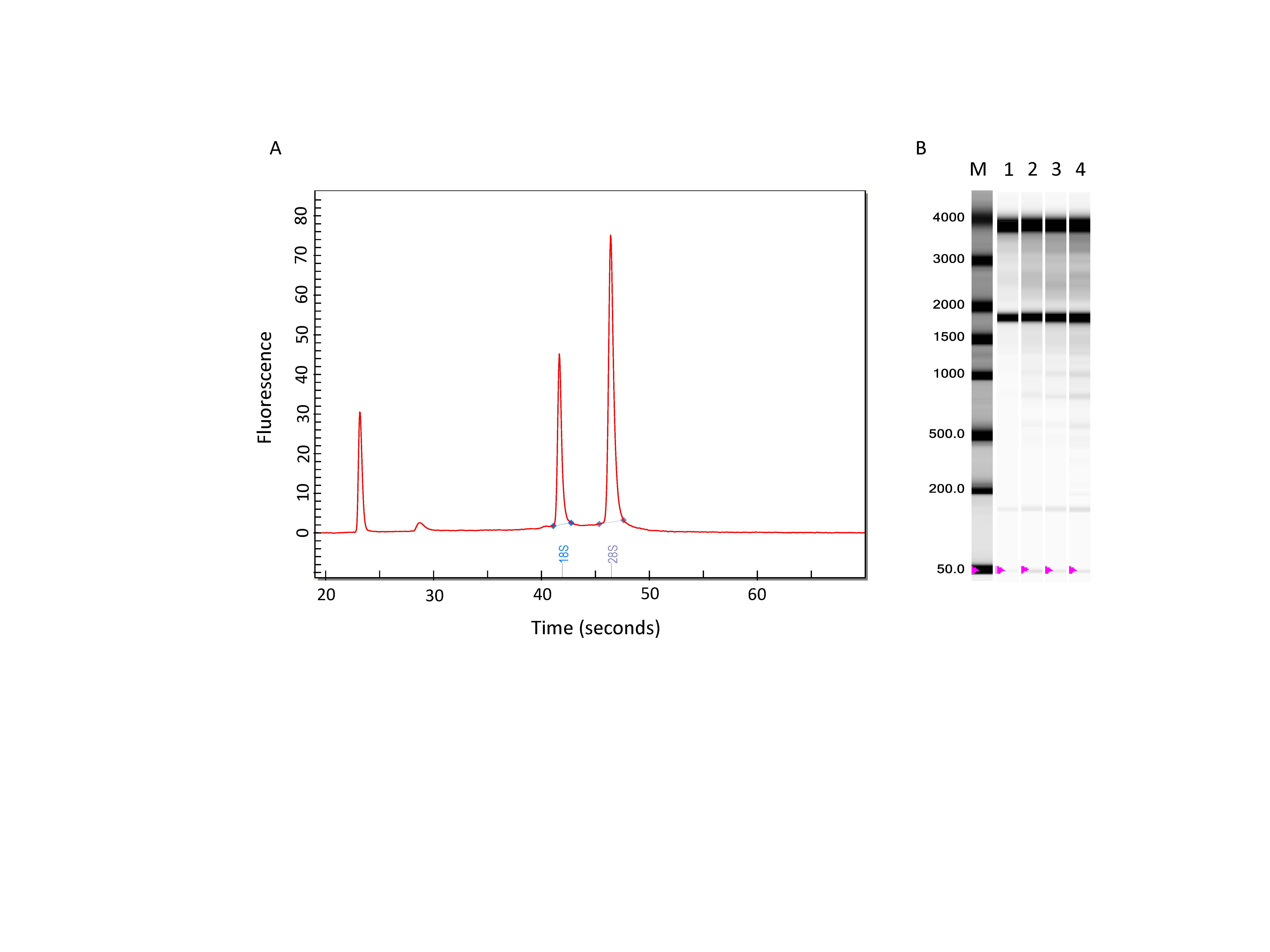

Supplement: Figure S1 — Total RNA samples were analyzed by the Experion Automated Electrophoresis System (Bio-Rad). Panel A shows a typical electropherogram of total RNA samples from Babesia bovis-infected bovine blood (The relative positions of 18S rRNA and 28S rRNA are indicated). Panel B shows a typical microfluidic electrophoresis of four representative RNA samples (1 to 4) and a molecular marker (M). The four representative samples presented RNA Quality Indicator (RQI) ≥ than 7 when analyzed by the Experion software, and in this study, only samples with RQI ≥ than 7 were used for cDNA synthesis. (TIFF) [file pone.0067765.s001.tiff]

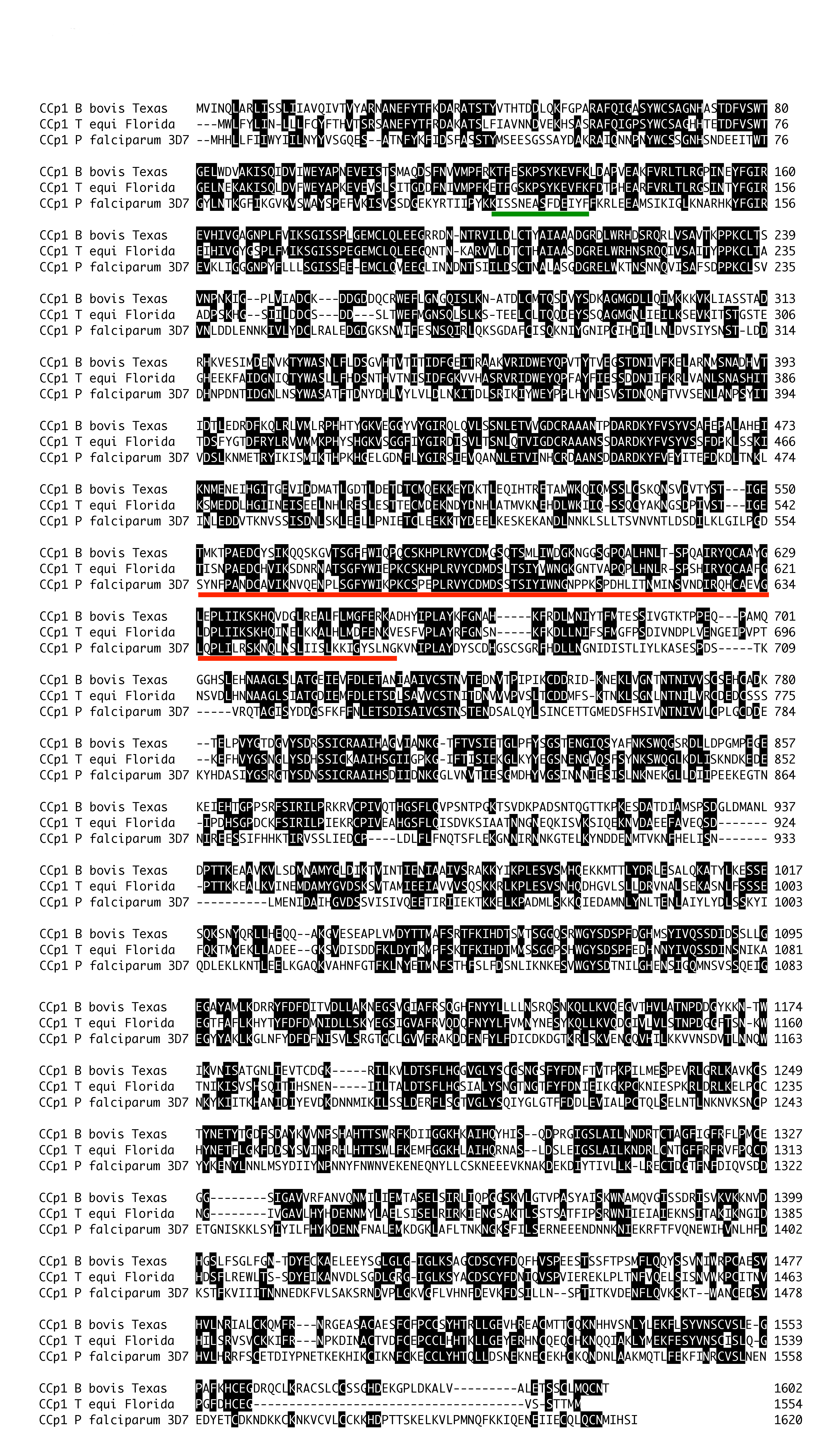

Supplement: Figure S2 — Multiple sequence alignments of CCp1 by CLUSTALW2. CCp2 amino acid sequences from Babesia bovis (Texas strain), Theileira equi (Florida isolate), Plasmodium falciparum (3D7 strain) and Plasmodium vivax (Sal-1 strain) were analyzed. Red line indicates the predicted location of the LCCL signature domains of the CCp protein family. Green line indicates the regions used to design synthetic peptides. (TIF) [file pone.0067765.s002.tif]

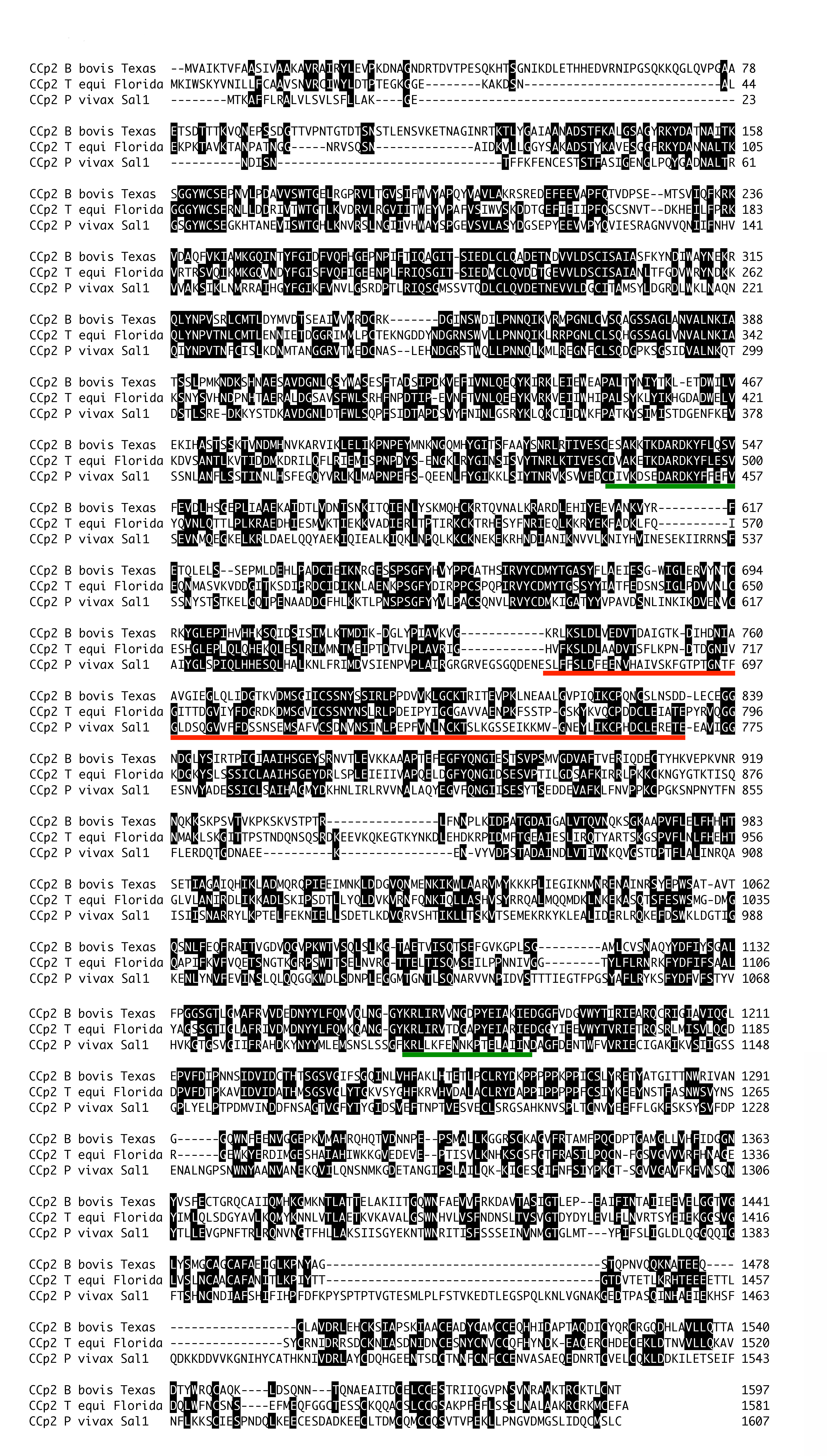

Supplement: Figure S3 — Multiple sequence alignments of CCp2 by CLUSTALW2. CCp3 amino acid sequences from Babesia bovis (Texas strain), Theileira equi (Florida isolate), Plasmodium falciparum (3D7 strain) and Plasmodium vivax (Sal-1 strain) were analyzed. Red line indicates the predicted location of the LCCL signature domains of the CCp protein family. Green lines indicate the regions used to design synthetic peptides. (TIF) [file pone.0067765.s003.tif]

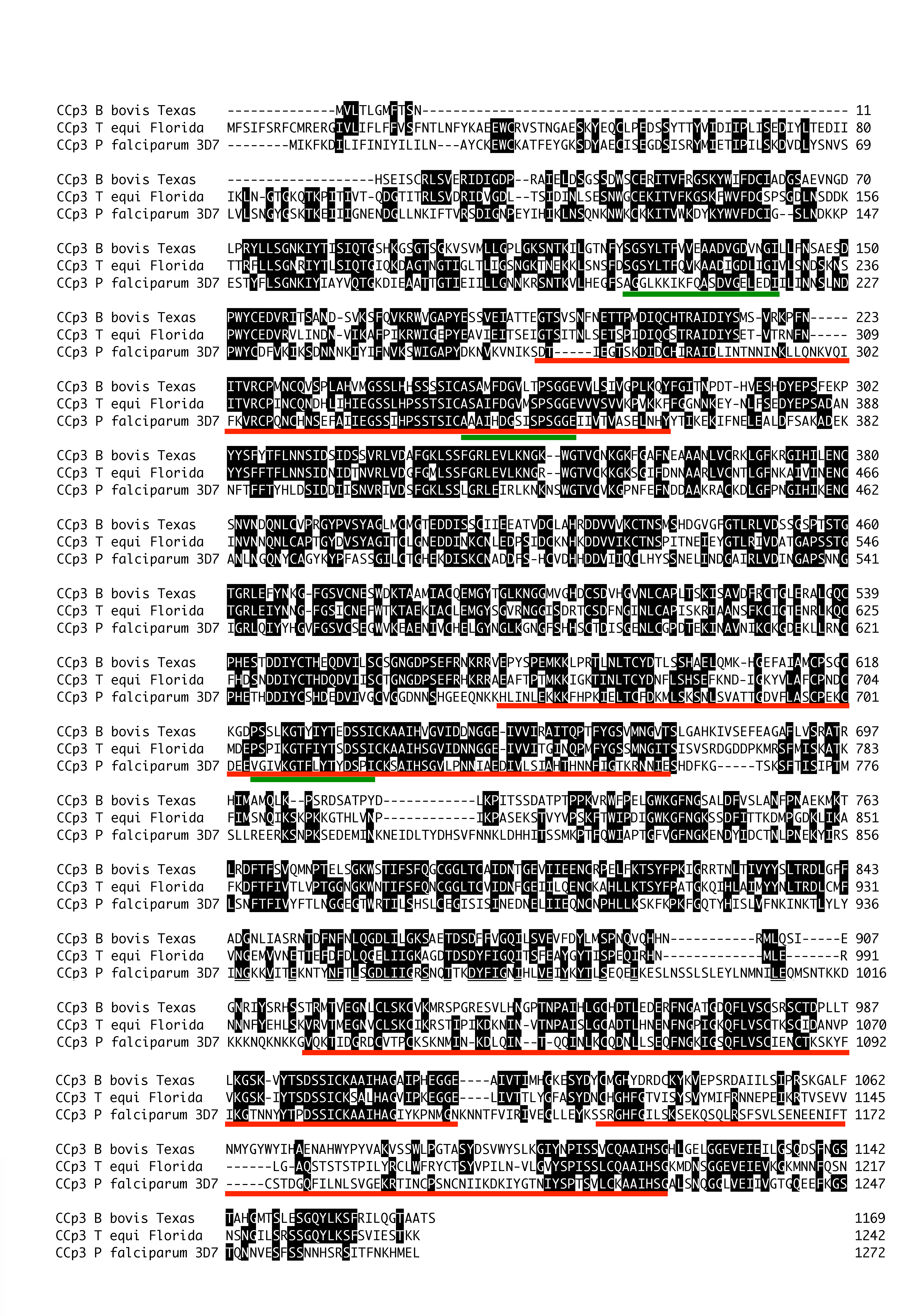

Supplement: Figure S4 — Multiple sequence alignments of CCp2 by CLUSTALW2. CCp3 amino acid sequences from Babesia bovis (Texas strain), Theileira equi (Florida isolate), Plasmodium falciparum (3D7 strain) and Plasmodium vivax (Sal-1 strain) were analyzed. Red lines indicate the predicted location of the LCCL signature domains of the CCp protein family. Green lines indicate the regions used to design synthetic peptides. (TIF) [file pone.0067765.s004.tif]

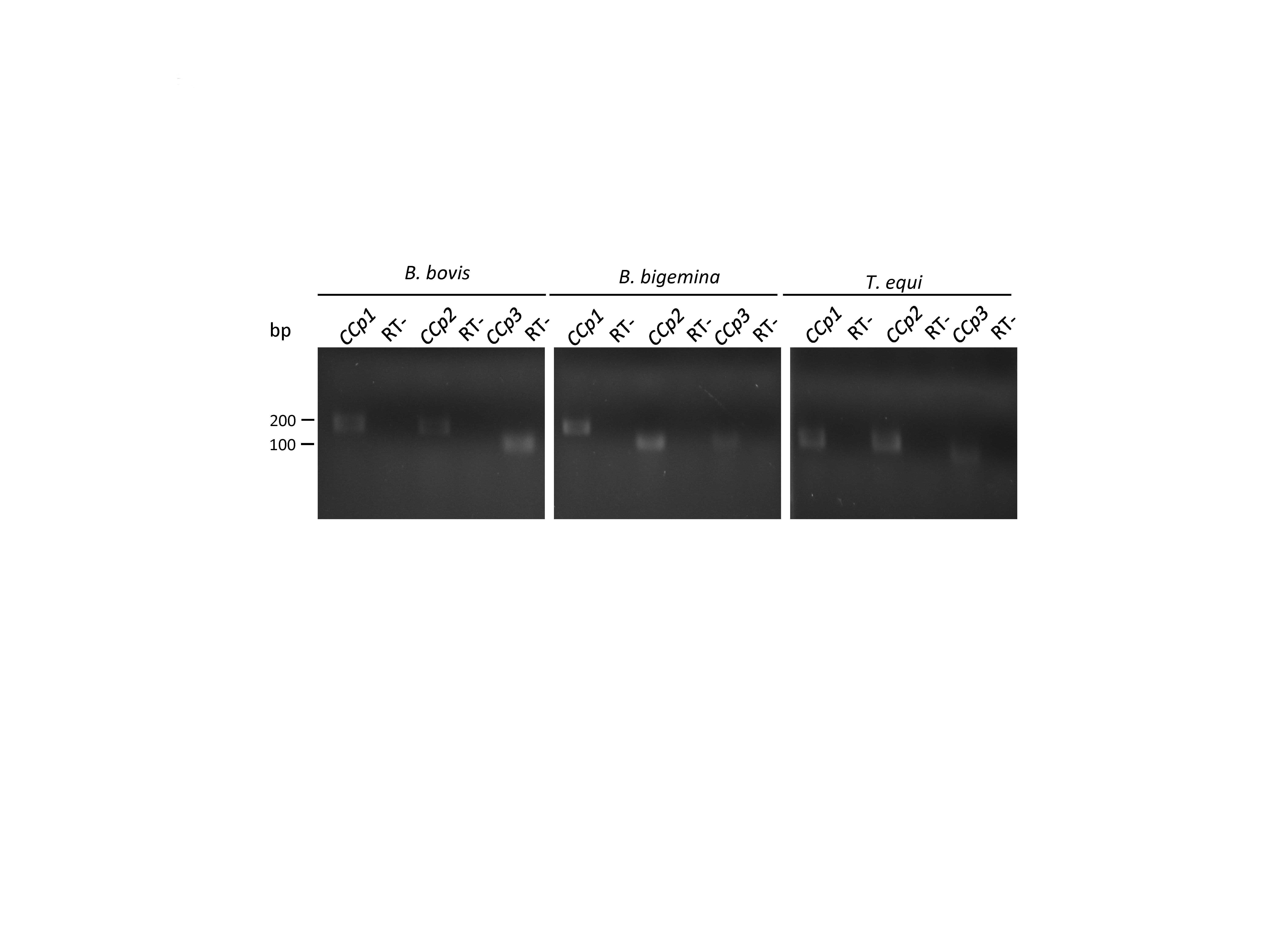

Supplement: Figure S5 — Presence of CCp1 - 3 transcripts in cultures of Babesia bovis , Babesia bigemina and Theileria equi kept at 37°C. Results without reverse transcriptase (RT-) are shown for each gene and parasite. (TIFF) [file pone.0067765.s005.tiff]

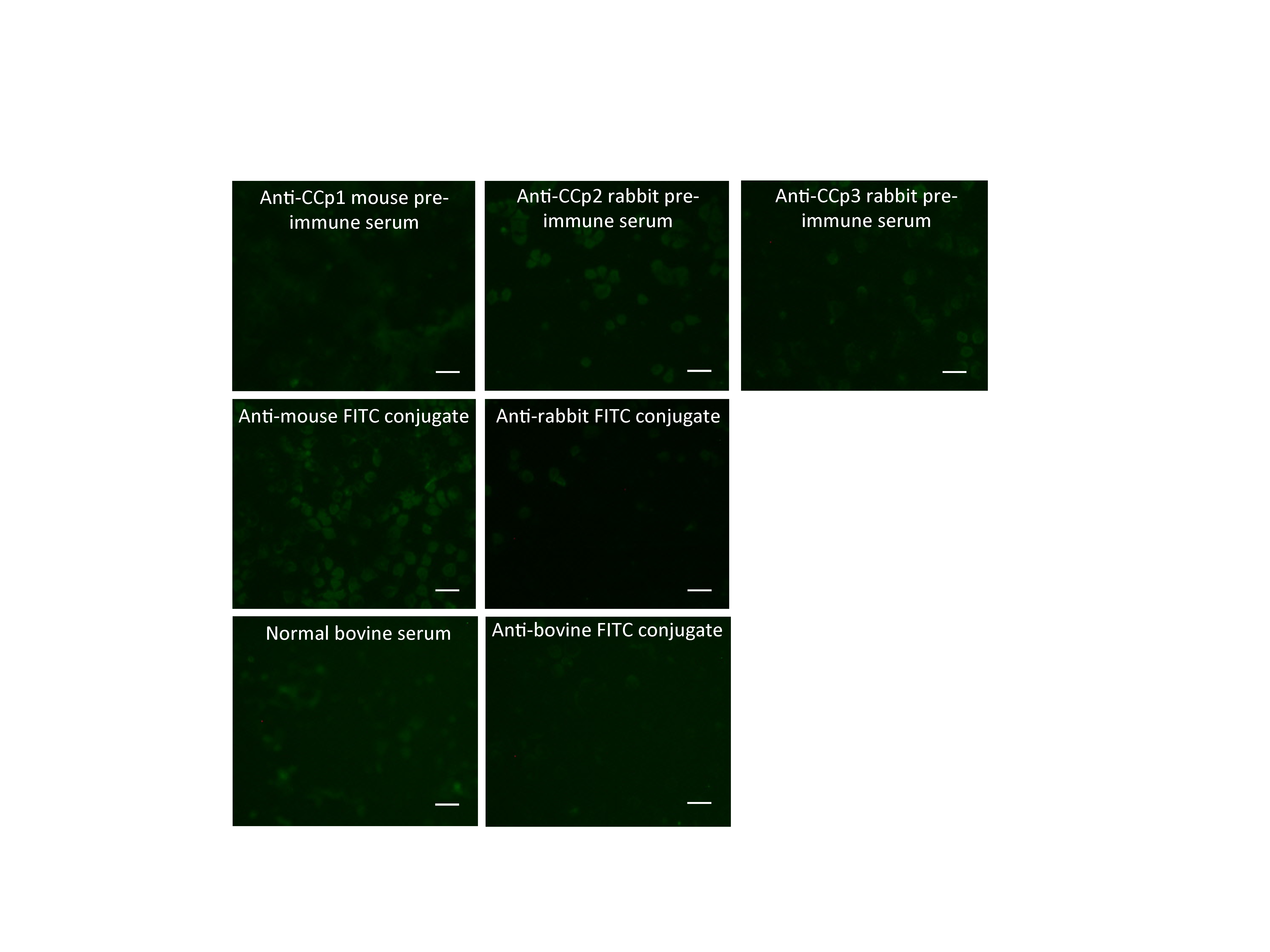

Supplement: Figure S6 — Immunofluorescence assays of pre-immune sera, normal bovine serum, anti-mouse FITC conjugate, anti-rabbit FITC conjugate, and anti-bovine FITC conjugate. Mouse and rabbit pre-immune sera were used at a 1∶20 dilution. Normal bovine sera were used at a 1∶20 dilution. Anti-mouse, anti-rabbit, or anti-bovine FITC conjugates were used at a 1∶80 dilution. The fields shown here are representative of the majority of the samples. The panels present a magnification of 100x and white bars indicate 10 µm. (TIFF) [file pone.0067765.s006.tiff]
