# Supplementary material for: Differential Expression of Three Members of the Multidomain Adhesion CCp Family in Babesia bigemina, Babesia bovis and Theileria equi
Source: PLoS One. 2013 Jul 3;8(7):e67765. doi: 10.1371/journal.pone.0067765 (PMC3701008; doi:10.1371/journal.pone.0067765)
Supplement: Table S1 — Gene name, primer sequence, amplicon size and parameters of PCR efficiency of the CCp genes in Babesia bovis ( Bb ), Babesia bigemina ( Bbg ) and Theileria equi ( Te ). (DOC) [file pone.0067765.s007.doc]

Table S1. Gene name, primer sequence, amplicon size and parameters of PCR efficiency of the *CCp* genes in *Babesia bovis* (*Bb*), *Babesia bigemina* (*Bbg*) and *Theileria equi* (*Te*).

| Gene name | Left and right primers | Amplicon size (bp) | Efficiency of amplification (%) | R^2^ | Slope |
| --- | --- | --- | --- | --- | --- |
| *BbCCp1* | tgtatggtattcgtcagtt and gtcgtctatcacttcacc | 200 | 108.5 | 0.95 | -3.13 |
| *BbCCp2* | ttagccgttgatagactt and ctgctgtggtttgtaatag | 178 | 102.6 | 0.99 | -3.26 |
| *BbCCp3* | cttcattacaccactcct and aagacaccatcaaacatag | 58 | 112.3 | 0.92 | -3.05 |
| *BbgCCp1* | atgttatgtatttcgtcttc and gagggtttgtgtatgga | 187 | 103.0 | 0.99 | -3.25 |
| *BbgCCp2* | gcgggttctactacatc and actcatccaacttacacg | 105 | 95.5 | 0.97 | -3.43 |
| *BbgCCp3* | tactcgttctacacattcc and aaaccaagcaaccttac | 84 | 101.9 | 0.98 | -3.27 |
| *TeCCp1* | acagcctttacacaatcta and attcaaccttattctcaaa | 149 | 103.4 | 0.99 | -3.24 |
| *TeCCp2* | aatctctgcctatctcaac and cttacagcactaccatcc | 137 | 102.3 | 0.99 | -3.20 |
| *TeCCp3* | gcatccatcttctacatc and ccactgagactacaacttc | 86 | 98.0 | 0.97 | -3.34 |
